# Smart device ownership and depressive symptoms among older adults: evidence from China

**DOI:** 10.3389/fpubh.2026.1881246

**Published:** 2026-08-06

**Authors:** Yanke Tong, Weihong Zeng, Pianpian Zhao

**Affiliations:** 1 Jinhe Center for Economic Research, Xi’an Jiaotong University, Xi'an, China; 2 School of Management, Xi’an University of Architecture and Technology, Xi'an, China

**Keywords:** depressive symptoms, digital inclusion, older adults, smart device ownership, social adaptation, social network

## Abstract

**Background:**

Against the backdrop of rapid digital technological advancement and the increasing diffusion of smart devices among older adults, understanding the implications of smart device for later-life well-being has become increasingly important. However, evidence remains limited on whether associations with depressive symptoms differ across types of smart devices and on the pathways linking smart device to depressive symptoms.

**Methods:**

Using pooled cross-sectional data from the 2018 and 2020 waves of the China Longitudinal Aging Social Survey (CLASS), this study estimated the association between smart device ownership and depressive symptoms among older adults using OLS regression models, with a final analytical sample of 12,269 observations. Sensitivity analyses were conducted using fixed-effects models, a 2020 subsample analysis, instrumental variable estimation, and propensity score matching. Mediation effect tests examined potential mechanisms involving social networks, offline activity participation, and social adaptation, while subgroup analyses were used to assess heterogeneity.

**Results:**

Smart device ownership was significantly associated with fewer depressive symptoms among older adults, with substantial variation across device functions and specific device types. Monitoring and assistive devices showed more consistent negative associations, whereas care devices showed the opposite pattern. This functional variation suggests that the association depends not only on whether older adults own smart devices, but also on the match between device functions and owners’ needs. Social networks and social adaptation appeared to be relevant pathways, and the negative association was more evident among older adults without functional limitations.

**Discussion:**

Functional differences across devices highlight the importance of aligning functions with older adults’ needs when considering the mental health implications of smart device ownership. As population aging deepens, age-friendly digital inclusion policies should move beyond an access-oriented perspective and place greater emphasis on matching device functions with older adults’ health needs, functional capacity, and social contexts.

## Introduction

Mental health, as a core dimension of healthy aging, profoundly influences older adults’ physical functioning, social participation, and well-being (1–4), while also generating spillover effects on families and health care systems (5). The mental health of older adults is likely shaped by a complex interplay of cognitive vulnerabilities, age-related neurobiological changes, and exposure to stressful life events (6, 7), therefore more vulnerable to psychological problems such as depression, anxiety, and loneliness (8). This vulnerability stems not only from individual-level factors, including physiological decline and the burden of chronic diseases (9), but also from family-level and society-level changes, such as weakening family support and shrinking social networks (10, 11), retirement (12), subjective social status (13), as well as internet use behavior (14, 15). Mental disorders such as depression and anxiety rank among the leading causes of mortality and disease burden worldwide (16), and evidence indicates that depression among adults aged 85 and above is significantly associated with an elevated risk of mortality within a two-year period (17), underscoring the critical importance of mental health research among older populations.

Among the social changes reshaping later-life well-being, the rapid expansion of digital technology has become particularly important for older adults’ daily lives (18, 19). In China, deepening population aging has made it increasingly important to examine how digital technology relates to older adults’ health and well-being (15). From the frailty perspective, older adults are especially vulnerable to digital adaptation pressure, as age-related declines in physiological, cognitive, and functional ability may limit their capacity to respond to rapidly changing digital environments (20, 21). Moreover, consistent with the ecological theory of aging, older adults’ psychological well-being may be undermined when environmental press exceeds their individual competence, creating a mismatch between personal capacity and the demands of the surrounding environment (22). While rapid digitalization may create “technostress” or “digital exclusion” when older adults’ skills, habits, and social roles fail to keep pace with the changing situation (23, 24), smart technologies may also enrich older adults’ spiritual and cultural lives by expanding access to cultural, recreational, and learning resources, thereby enhancing life meaning and well-being (25). This duality shows the complicated welfare implications of smart technologies in later life, as they may generate stress while also providing resources that support healthy ageing. Existing studies have therefore paid growing attention to the relationship between smart device and older adults’ mental health (26–29). Devices such as smartphones, tablets, and wearable technologies are no longer merely communication tools, but also key channels for accessing information, maintaining social connections, obtaining health-related services, and engaging in leisure activities (26). Current studies show that the accessibility, application and expansibility of family level smart devices have a significant positive impact on the life satisfaction of older adults (27). Some studies find that smart devices are associated with lower depression levels through expanding social connections, enhancing a sense of control over self-health management, and improving access to health services (28, 29).

At the same time, however, older adults may face barriers to smart device access, digital exclusion, and difficulties adapting to rapidly evolving digital environments, which may generate frustration, stress, and a sense of marginalization (30). Therefore, whether smart device ultimately mitigates or exacerbates depression among older adults remains an open question. On the one hand, some studies find that digital technologies, including computers, smartphones, smartwatches, and health-tracking apps, is not associated with depression or anxiety, but is instead significantly related to factors such as age, insurance coverage, socioeconomic status, and physical activity (31). On the other hand, evidence shows that the excessive use of smartphones may contribute to psychological stress of older adults (32). In addition, the benefits of digitalization are not evenly distributed among older population, most of the studies mentioned above report heterogeneous effects across population groups and usage contexts, and disadvantaged groups such as rural older migrant women tend to benefit less (33).

Existing studies examining the mechanisms through which digitalization affects older adults’ mental health typically focus on factors such as the quantity and strength of social capital (34), physical exercise (35), participation in leisure activities (12), education level (36), and loneliness (37). These studies suggest that digitalization influence mental health by shaping daily routines, social interactions, and lifestyle patterns among older adults. Moreover, the widespread adoption of digital technologies has introduced attitudinal changes among older adults, which serve as important mechanisms. These mechanisms include shifts in attitudes toward aging (38), perceptions of social fairness and political participation (39), the allocation of time between screen time and family interactions (40), as well as self-efficacy for managing health (41).

As shown above, while the literature on digitalization and older adults’ mental health highlights the potential of digital technologies to promote smart aging and well-being, research explicitly examining broader dimensions of smart device remains limited. Existing studies often adopt a narrow perspective by focusing primarily on communication device ownership, general internet access, or a single type of device. Insufficient attention has been paid to the breadth of smart device ownership, functional differences across device categories, and specific device heterogeneity. Moreover, the mechanisms linking smart devices to older adults’ mental health remain insufficiently examined.

This study makes three main contributions to current research. First, unlike studies that focus on general indicators of smart device ownership, this paper distinguishes whether older adults own smart devices, how many types they own, and which functional categories and specific device types they own. This more detailed measurement captures variation across device functions and specific device types. Second, this study highlights the functional meaning of smart device ownership by considering how different devices correspond to older adults’ health-monitoring, assistive, and care needs. This perspective helps explain why the association with depressive symptoms may differ across device categories. Third, this study examines potential pathways through social networks, offline activity, and social adaptation, and assesses subgroup differences by living arrangement, age, and functional limitation.

The remainder of this paper is organized as follows. Section 2 presents the data, variables, and empirical strategy. Section 3 reports the descriptive statistics and baseline results. Section 4 conducts sensitivity analyses and further explores the mechanisms and subgroup differences. Section 5 concludes with a summary of the main findings, limitations, and policy implications.

## Data, variables, and empirical strategy

### Data source

Our study draws on data from the 2018 and 2020 waves of the Chinese Longitudinal Aging Social Survey (CLASS). The CLASS is a large-scale, nationally representative longitudinal survey conducted by the China Survey and Data Center at Renmin University of China. It covers 28 provinces, excluding Hainan, Tibet, and Xinjiang, and provides rich information on the socioeconomic status, health conditions, family structures, and social support networks of older adults across diverse regions in China. In this study, the two waves were pooled for empirical analysis.

After pooling the 2018 and 2020 waves, the initial sample consisted of 22,811 observations, including 11,416 observations from 2018 and 11,395 from 2020. We excluded observations with missing values in key variables, resulting in the removal of 10,542 observations. The final analytical sample comprised 12,269 observations.

### Variables

#### Dependent variable

The dependent variable in this study is an index of depressive symptoms constructed from 9 self-reported items capturing respondents’ emotional well-being during the past week. Specifically, respondents were asked whether they (1) felt in a good mood, (2) felt lonely, (3) felt sad, (4) felt that life was going well, (5) had a poor appetite, (6) experienced sleep problems, (7) felt useless, (8) felt that they had nothing to do, and (9) felt that there was much enjoyment in life. Each item was recoded on a three-point scale, with 0 indicating “not at all,” 1 indicating “sometimes,” and 2 indicating “often.” Following standard practice in the literature on mental health measurement among older adults, responses to positively worded items were reverse-coded so that higher values consistently indicate more severe depressive symptoms. The nine items were then summed to generate a composite score ranging from 0 to 18, with higher scores representing worse mental health status. This index captures the respondent’s emotional distress, loneliness, somatic symptoms, perceived worthlessness, lack of purpose, and reduced enjoyment of life.

#### Independent variable

This study uses smart device ownership as the core independent variable, since the CLASS survey identifies ownership of specific smart devices rather than detailed usage behavior. This ownership-based measure captures not only access to these devices, but also the presence of specific functional resources that may be relevant to depressive symptoms among older adults. Specifically, the questionnaire asks respondents whether they have each type of smart device, separately. Given that different devices serve distinct purposes and may reflect health-monitoring, assistive, or care-related needs of their owners, we classified the eight smart devices into three functional categories:

1. Smart monitoring devices include electronic blood pressure monitor, cholesterol device, smart bandwatch, and sleep monitor. The primary function of these devices is to support the monitoring of physiological indicators.
2. Smart assistive devices include smart wheelchair and audiobook device. This category provides functional assistance, or compensatory support for sensory and activity limitations in daily life.
3. Smart care devices include smart camera and smart speaker. This category is mainly designed for household assistance, communication, or care-related interaction, and may therefore reflect care needs as well as family connection.

We measure smart device ownership using several related indicators. The extensive margin is captured by a binary indicator that takes the value of 1 if the respondent owns any of the eight types of smart devices, and 0 otherwise. We also include the total number of smart device types owned by the respondent, as well as indicators for functional categories and specific device types to capture heterogeneity in device functions and the needs associated with ownership.

#### Control variables

We control for a set of demographic, health, family and social resource covariates commonly identified in the literature as factors associated with older adults’ depressive symptoms. These variables can be grouped into four categories as follows: (1) Demographic characteristics include gender, age, marital status, educational level, hukou status, employment status, and income. (2) Health behavior and status include smoking behavior, cognitive level, the presence of chronic diseases, and limitations in activities of daily living (ADL). (3) Family information includes the number of children and living arrangements. (4) Social resource and security level include whether the respondent used any community healthcare services in the past 12 months, whether the respondent was hospitalized in the past 2 years, and social security benefits.

#### Mediating variable

Given the substantial heterogeneity in social participation, daily activity, and health needs across different stages of ageing (42), this paper examines three potential channels through which smart device ownership may be associated with depressive symptoms: social networks, offline activity, and social adaptation.

Social network. Existing studies have shown that more frequent social contact is positively associated with successful and robust ageing (43), and both the quantity and quality of social relationships have significant effects on mental health and psychological well-being across the life course (34, 44). Smart device ownership may indicate potential opportunities for instrumental and emotional communication and may therefore be associated with better perceived and actual social support, which is in turn related to lower social isolation and fewer depressive symptoms. In this study, social network was measured based on respondents’ social connections and perceived support from family members, relatives, and friends. Respondents reported the number of people with whom they maintained regular contact, could discuss private matters, or could rely on for help when needed. Each item was coded on a six-point scale ranging from 0 to 5, and a composite social network index was constructed by summing the six items, with higher scores indicating broader social networks and stronger perceived social support.

Offline activity. Smart devices may be associated with fewer constraints on daily activities and broader offline participation, especially when these devices are linked to perceived safety, mobility, and health management. This may increase opportunities for older adults to engage in outdoor activities such as volunteering and group leisure activities, which are in turn associated with better subjective well-being, and higher life satisfaction (45–47). In this study, this variable was measured based on respondents’ self-reported frequency of participation in offline activities over the past year, including (1) religious activities, (2) attending training courses, (3) watching television, listening radios, reading books or newspapers, (4) listening opera, singing or playing musical instruments, (5) playing mahjong, chess, or cards, and (6) participating in square dancing, which is a popular outdoor group dance among older adults in China. Responses were coded on a five-point scale ranging from 0 to 4, and a composite offline activity index was constructed by summing the six items, with higher scores indicating greater engagement in offline activities.

Social adaptation. By indicating potential access to digital-related products and services, smart devices may be associated with better adaptation to social change and strengthen their perceived social inclusion (48). Beyond its association with activity participation, smart device ownership may also be linked to older adults’ confidence in navigating an increasingly digital society and to more positive attitudes toward social change, which may reflect adaptive capabilities associated with fewer depressive symptoms (29). In this study, this variable was measured based on respondents’ self-reported attitudes toward social adaptation capacity, including (1) willingness to engage in local public affairs, (2) desire to contribute to society, (3) enjoyment of learning, (4) acceptance of social change, (5) acceptance of new views, (6) acceptance of new social policies, and (7) perceived inclusiveness of social change for older adults. Responses were coded on a five-point scale, and a composite social adaptation index was constructed by summing these items, with higher scores indicate stronger social adaptation and orientation.

### Empirical strategy

To estimate the association between smart device ownership and older adults’ depression symptom scores, we begin with the following baseline models (see Equation 1):

$$\;\text{Depressio}n_{i}=\alpha +\beta D_{i}+\text{γContro}l_{i}+\varepsilon_{i}$$ (1)

Where $\text{Depressio}n_{i}$ denotes depressive symptom scores, $D_{i}$ captures smart device ownership, and $\text{Contro}l_{i}$ is a vector of control variables.

Rather than treating smart device ownership as unidimensional construct, this study conceptualizes it along four empirical dimensions. This multidimensional measurement strategy allows us to distinguish basic device ownership, the breadth of ownership, functional categories, and heterogeneity across specific device types (see Equations 2–5):

1. Extensive Margin

We first consider whether older adults own any smart device, measured by if_smart_devicei. This dimension captures the extensive margin of smart device ownership and reflects whether the respondent has access to at least one smart device.

$$\text{Depressio}n_{i}=\alpha +\mathrm{\beta if}\_\text{smart}\_\text{devic}e_{i}+\text{γContro}l_{i}+\varepsilon_{i}$$ (2)

2. Breadth Margin

We measure the total number of smart device types owned by the respondent using $\mathrm{HW}\_\text{tota}l_{i}$ , where HW denotes hardware smart devices. This variable captures the breadth of smart device ownership, reflecting the range of smart device functions available to older adults in daily life.

$$\text{Depressio}n_{i}=\alpha +\mathrm{\beta HW}\_\text{tota}l_{i}+\text{γContro}l_{i}+\varepsilon_{i}$$ (3)

3. Functional Categories

We clarified smart devices into three functional categories, denoted as $\mathrm{HW}\_{\text{monitor}}_{i}$ , $\mathrm{HW}\_{\text{assist}}_{i}$ , and $\mathrm{HW}\_{\text{care}}_{i}$ . This dimension captures the functional meaning of smart device ownership by distinguishing health monitoring, assistance, and care contexts, thereby reflecting the functional differences among the devices owned by respondents.

$$\begin{array}{c} \text{Depressio}n_{i}=\alpha +\beta_{1}\mathrm{HW}\_\text{monito}r_{i}+\beta_{2}\mathrm{HW}\_\text{assis}t_{i}\\ +\beta_{3}\mathrm{HW}\_\mathrm{car}e_{i}+\text{γContro}l_{i}+\varepsilon_{i}\; \end{array}$$ (4)

4. Device Heterogeneity

This study further distinguishes eight specific types of smart device to examine whether the association varies across specific devices.

$$\begin{array}{c} \text{Depressio}n_{i}=\alpha +\beta_{1}\text{smart}\_\text{wheelchai}r_{i}+\beta_{2}\text{elec}\_bp_{i}\\ +\beta_{3}\text{cholesterol}\_\text{devic}e_{i}+\beta_{4}\text{smart}\_\text{bandwatc}h_{i}\\ +\beta_{5}\text{smart}\_\text{camer}a_{i}+\beta_{6}\text{ssmart}\_\text{speake}r_{i}\\ +\beta_{7}\text{sleep}\_\text{monito}r_{i}+\beta_{8}\text{audiobook}\_\text{devic}e_{i}\\ +\text{γContro}l_{i}+\varepsilon_{i} \end{array}$$ (5)

These four dimensions provide a more granular framework for examining the extensive margin, breadth margin, functional categories, and device-specific heterogeneity of smart device ownership. Each specification is estimated separately to reduce multicollinearity concerns and to better identify the association captured by each dimension. These models are estimated using pooled OLS with robust standard errors. All analysis has been conducted with Stata 17 (Table 1).

**Table 1:** Definition of variables.

Variables	Definition
Dependent variable	
Depression	Sum of scores from the 9-item depression scale (good mood, lonely, sad, life going well, poor appetite, sleep problems, useless, nothing to do, enjoyment).
Independent variable	
If_smart_device	Equals 1 if the respondent owns any of the eight smart devices, and 0 otherwise.
HW_total	Number of smart devices the respondent owned.
HW_monitor	Equals 1 if the respondent owns smart monitoring devices, and 0 otherwise.
HW_assist	Equals 1 if the respondent owns smart assistive devices, and 0 otherwise.
HW_care	Equals 1 if the respondent owns smart care devices, and 0 otherwise.
Smart Wheelchair	Equals 1 if the respondent owns a smart wheelchair, and 0 otherwise.
Elec_bp	Equals 1 if the respondent owns an electronic blood pressure monitor, and 0 otherwise.
Cholesterol Device	Equals 1 if the respondent owns a cholesterol device, and 0 otherwise.
Smart Bandwatch	Equals 1 if the respondent owns a smart bandwatch, and 0 otherwise.
Smart Camera	Equals 1 if the respondent owns a smart camera, and 0 otherwise.
Smart Speaker	Equals 1 if the respondent owns a smart speaker, and 0 otherwise.
Sleep Monitor	Equals 1 if the respondent owns a smart sleep monitor, and 0 otherwise.
Audiobook Device	Equals 1 if the respondent owns an audiobook device, and 0 otherwise.
Demographic characteristics	
Gender	Equals 1 if the respondent is male; 0 if female.
Age	Respondent’s age
Marriage	Equals 1 if the respondent is married with a spouse, and 0 otherwise.
Education Level	Equals 1 if the respondent finished primary education, and 0 otherwise.
Rural	Equals 1 if the respondent has an agricultural household registration, and 0 otherwise.
Job Situation	Equals 1 if the respondent is currently employed, and 0 otherwise.
Lnincome	Logarithm of respondent’s total income.
Health behavior and status	
Smoke	Equals 1 if the respondent smokes, and 0 otherwise
Cognitive Level	Respondent’s cognitive ability score.
Chronic Disease	Equals 1 if the respondent has any chronic disease (e.g., heart attack, high blood pressure, stroke, diabetes, or lung disease), and 0 otherwise.
ADL	Sum of scores for six activities of daily living (bathing, dressing, toileting, transferring, walking, eating).
Family information	
Children	Number of children.
Living Arrangement	Number of people living with the respondent, including the respondent.
Social resource and security level	
Any Healthcare Utilization	Whether the respondent used any community healthcare services in the past 12 months.
Any Hospitalization	Whether the respondent was hospitalized in the past two years.
Social Security	Total amount of social security benefits received by the respondent.
Mediating variable	
Social Network	Sum of scores for social support and network from family members, relatives, and friends (regular contact, private discussion, help availability).
Offline Activity	Sum of scores for offline activities (religious, courses, television/radio/reading, opera/musical instruments, mahjong/chess/cards, dancing).
Social Adaptation	Sum of scores for social adaptation and social orientation (public-affairs engagement, society contribution, enjoy learning, social change, new views, new policies, perceived inclusiveness).
Instrument variable	
Internet Signal	Equals 1 if the respondent’s residence has internet signal, and 0 otherwise.

## Descriptive statistics and baseline results

### Descriptive statistics

Table 2 reports descriptive statistics for the full analytical sample and by smart device ownership status. The final sample includes 12,269 observations, of which 5,028 (41.0%) reported own at least one smart device. The mean depressive symptom scores were 6.527 in the full sample. The average age was about 71 years old, 72.7% of the respondents were married or cohabited, 44.1% had attained primary education or above, and 37.2% held rural hukou status. The mean cognitive score was 13.456 on a 0–16 scale, 78.6% reported at least one chronic disease, and the average ADL limitation count was 0.182. About 33.3% of respondents reported community healthcare utilization in past 12 months, and 26.7% reported hospitalization in past 2 years.

**Table 2:** Descriptive statistics of the variables.

Variables	Full sample (*N* = 12,269)		With smart device (*N* = 5,028)		No smart device (*N* = 7,241)		Mean test
	Mean	SD	Mean	SD	Mean	SD	
If_smart_device	0.410	0.492					
Depression	6.527	3.176	6.098	3.182	6.825	3.138	***
Gender							
Female	0.506	0.500	0.484	0.500	0.522	0.500	***
Male	0.494	0.500	0.516	0.500	0.478	0.500	***
Age	71.172	7.073	71.227	7.143	71.134	7.025	
Marital status							
Single, separated, widowed or divorced	0.273	0.445	0.239	0.427	0.296	0.456	***
Married or cohabiting	0.727	0.445	0.761	0.427	0.704	0.456	***
Education level							
> = Primary education	0.441	0.497	0.573	0.495	0.349	0.477	***
<Primary education	0.559	0.497	0.427	0.495	0.651	0.477	***
Rural							
Yes	0.372	0.483	0.190	0.392	0.499	0.500	***
No	0.628	0.483	0.810	0.392	0.501	0.500	***
Job Situation							
Not in work	0.818	0.386	0.915	0.279	0.751	0.433	***
In work	0.182	0.386	0.085	0.279	0.249	0.433	***
Lnincome	8.439	1.339	8.957	1.083	8.080	1.382	***
Smoke							
Yes	0.317	0.465	0.327	0.469	0.310	0.462	**
No	0.683	0.465	0.673	0.469	0.690	0.462	**
Cognitive level	13.456	3.101	13.697	2.790	13.288	3.289	***
Chronic disease							
Yes	0.786	0.410	0.830	0.376	0.755	0.430	***
No	0.214	0.410	0.170	0.376	0.245	0.430	***
ADL	0.182	0.808	0.239	0.924	0.142	0.713	***
Children	2.358	1.326	2.063	1.204	2.562	1.368	***
Living Arrangement	2.623	1.235	2.698	1.236	2.570	1.232	***
Any Healthcare Utilization	0.333	0.471	0.347	0.476	0.324	0.468	***
Any Hospitalization	0.267	0.443	0.283	0.450	0.256	0.437	***
Social Security	7.182	2.727	7.997	2.415	6.616	2.788	***

We conducted a mean comparison test for variables between older adults who owned and did not own smart devices. See Table 1 for the complete definitions of all variables. * *p* < 0.10; ** *p* < 0.05; *** *p* < 0.01.

Compared with smart device non-owners, older adults who own smart devices had a lower mean depressive symptom score than non-owners, with average scores of 6.098 and 6.825, respectively. Owners were more likely to be married or cohabiting, better educated, hold urban hukou status, have higher income and social security benefits, and exhibit better cognitive function. They also reported a higher prevalence of chronic diseases, greater limitations in activities of daily living (ADL), and more frequent utilization of community healthcare services and hospitalization.

### Baseline results

We estimate pooled OLS models to examine the association between smart device ownership and depressive symptoms among older adults. The results are shown in Table 3 and Appendix Figure A1. Model (1) examines the association between any smart device ownership and depressive symptoms. Model (2) assesses the breadth of ownership using the total number of smart device types owned. Model (3) further differentiates ownership by functional categories of smart devices, while Model (4) estimates the associations for each specific device type separately.

**Table 3:** OLS regression estimates of the association between smart device ownership and depressive symptoms among older adults.

Variables	(1)	(2)	(3)	(4)
	Any ownership	Number of devices owned	By category	By device
If_smart_device	−0.496***			
	[−0.617, −0.375]			
HW_total		−0.238***		
		[−0.291, −0.185]		
HW_monitor			−0.317***	
			[−0.395, −0.240]	
HW_assist			−0.505***	
			[−0.719, −0.292]	
HW_care			0.538***	
			[0.329, 0.747]	
Smart Wheelchair				0.646***
				[0.332, 0.960]
Elec_bp				−0.461***
				[−0.590, −0.332]
Cholesterol device				0.280***
				[0.081, 0.478]
Smart bandwatch				−0.577***
				[−0.809, −0.345]
Smart camera				−0.447*
				[−0.947, 0.054]
Smart speaker				0.989***
				[0.764, 1.214]
Sleep monitor				−1.449***
				[−1.842, −1.055]
Audiobook device				−1.168***
				[−1.475, −0.862]
Demographic characteristics				
Gender	−0.062	−0.050	−0.045	−0.054
	[−0.184, 0.060]	[−0.171, 0.072]	[−0.167, 0.077]	[−0.175, 0.067]
Age	0.033***	0.033***	0.034***	0.032***
	[0.024, 0.042]	[0.024, 0.042]	[0.025, 0.044]	[0.022, 0.041]
Marital status	−0.466***	−0.465***	−0.464***	−0.457***
	[−0.595, −0.336]	[−0.594, −0.335]	[−0.593, −0.335]	[−0.585, −0.329]
Education level	−0.430***	−0.425***	−0.447***	−0.427***
	[−0.556, −0.304]	[−0.551, −0.299]	[−0.573, −0.321]	[−0.552, −0.302]
Rural	−0.453***	−0.463***	−0.456***	−0.432***
	[−0.608, −0.298]	[−0.619, −0.308]	[−0.611, −0.302]	[−0.587, −0.278]
Job situation	0.171**	0.185**	0.184**	0.183**
	[0.019, 0.323]	[0.034, 0.337]	[0.033, 0.336]	[0.032, 0.335]
Lnincome	−0.299***	−0.302***	−0.291***	−0.287***
	[−0.349, −0.250]	[−0.351, −0.253]	[−0.341, −0.242]	[−0.337, −0.238]
Health behavior and status				
Smoke	0.106	0.079	0.072	0.091
	[−0.021, 0.232]	[−0.048, 0.206]	[−0.055, 0.199]	[−0.035, 0.217]
Cognitive level	−0.059***	−0.055***	−0.056***	−0.058***
	[−0.079, −0.039]	[−0.075, −0.035]	[−0.076, −0.036]	[−0.077, −0.038]
Chronic disease	0.575***	0.585***	0.608***	0.603***
	[0.442, 0.709]	[0.451, 0.719]	[0.475, 0.742]	[0.470, 0.736]
ADL	0.338***	0.360***	0.373***	0.317***
	[0.262, 0.414]	[0.283, 0.437]	[0.296, 0.450]	[0.239, 0.395]
Family information				
Children	−0.046*	−0.048*	−0.047*	−0.047*
	[−0.095, 0.002]	[−0.096, 0.000]	[−0.094, 0.001]	[−0.095, 0.001]
Living arrangement	0.015	0.019	0.019	0.024
	[−0.030, 0.059]	[−0.025, 0.063]	[−0.026, 0.063]	[−0.020, 0.068]
Social resource and security level				
Any healthcare utilization	0.048	0.042	0.057	0.056
	[−0.069, 0.165]	[−0.075, 0.159]	[−0.060, 0.174]	[−0.060, 0.173]
Any hospitalization	0.750***	0.759***	0.769***	0.724***
	[0.626, 0.875]	[0.634, 0.883]	[0.645, 0.893]	[0.600, 0.848]
Social security	−0.045***	−0.044***	−0.044***	−0.047***
	[−0.070, −0.020]	[−0.069, −0.019]	[−0.069, −0.019]	[−0.071, −0.022]
Constant	8.034***	7.937***	7.764***	7.944***
	[7.183, 8.885]	[7.084, 8.790]	[6.912, 8.616]	[7.095, 8.793]
N	12,269	12,269	12,269	12,269
Adjusted R-squared	0.106	0.107	0.111	0.123

Outcome: depressive symptom score (higher = worse). All models include the same control set. See Table 1 for the complete definitions of all variables. Confidence intervals are reported in brackets. *p < 0.10; **p < 0.05; ***p < 0.01.

Table 3 shows that smart device ownership is significantly associated with lower depressive symptom scores. In model (1), older adults who owned at least one smart device had depressive symptom scores that were 0.496 points lower than those of non-owners ( 95%CI:[−0.617,−0.375] ). The reduction is equivalent to approximately 7.60% of the sample mean and 0.156 of the sample standard deviation. This estimate indicates a modest but meaningful difference in depressive symptom burden, as it reflects a lower overall level of depressive symptoms among older adults who own smart devices. Model (2) shows that each additional type of smart device owned was associated with a 0.238 points reduction in depressive symptom scores (95%CI:[−0.291,−0.185]) . The results in Model (3) show substantial heterogeneity across device functions. Monitoring and assistive devices are negatively associated with depressive symptoms (β=−0.317;95%CI:[−0.395,−0.240];β=−0.505;95%CI:[−0.719,−0.292]) , whereas smart care devices show the opposite pattern (β=0.538,95%CI:[0.329,0.747]) . This interesting finding implies that monitoring and assistive devices are more closely related to health management and functional support, while care devices may reflect greater underlying health vulnerability and care needs among their owners.

At the device level, Model (4) further reveals clear device-specific heterogeneity, with the corresponding coefficients and 95% confidence intervals reported in Table 3. Electronic blood pressure monitors, smart bandwatches, smart cameras, sleep monitors, and audiobook devices show negative associations with depressive symptoms. These devices might be more closely linked to routine health tracking, safety monitoring, sleep management, and improved access to information or cultural resources for older adults with reading difficulties, thereby contributing to potential psychological benefits. By contrast, smart wheelchairs, cholesterol devices, and smart speakers show positive associations. These differences suggest that device-specific associations partly reflect the health needs and contexts in which devices are owned and potentially used. The positive coefficients of smart wheelchairs and cholesterol devices ownership may indicate mobility limitations or disease-management needs, while smart speakers may reflect higher operational barriers or may be purchased by older adults with greater care and companionship needs.

Regarding covariates, the coefficients, 95% confidence intervals, and *p*-values are reported in Table 3. Depressive symptom scores increase with age and are higher among respondents with chronic diseases, ADL limitations, and recent hospitalization. Being married or cohabiting, having higher education, higher income, higher social security benefits, and higher cognitive scores are consistently associated with lower depressive symptom scores. Community healthcare utilization is not statistically significant, which may reflect the limited or uneven use of community-based healthcare services among older adults in China. These patterns are broadly consistent with existing evidence that later-life mental health is shaped by health status, functional capacity, cognitive resources, family support, and socioeconomic conditions (6–13).

## Further analysis

### Sensitivity analyses

To examine the robustness of our baseline estimates, we perform a series of sensitivity analyses. Table 4 reports alternative model specifications, including individual fixed-effects models, OLS estimates based on the 2020 sample, and 2SLS estimates using internet signal access as an instrumental variable. These analyses assess whether the baseline associations are sensitive to time-invariant unobserved heterogeneity, sample composition, and potential endogeneity. In addition, we apply same-year propensity score matching to balance observable characteristics between owners and non-owners of smart devices, with the results reported in Table 5.

**Table 4:** Sensitivity analysis.

Variables	(1)	(2)	(3)
	FE	OLS (2020)	IV (2SLS)
Panel a: impact of any smart device ownership			
If_smart_device	−0.233	−0.449***	−5.295***
	[−0.610, 0.144]	[−0.664, −0.234]	[−6.584, −4.007]
Observations	12,269	3,989	12,269
Adjusted R-squared	0.062	0.108	
First-Stage F-Stat			150.322
Panel b: impact of number of smart devices owned			
HW_total	−0.208***	−0.189***	−2.074***
	[−0.332, −0.084]	[−0.268, −0.110]	[−2.552, −1.596]
Observations	12,269	3,989	12,269
Adjusted R-squared	0.068	0.109	
First-Stage F-Stat			212.988

Outcome: depressive symptom score (higher = worse). All models include the same control set. See Table 1 for the complete definitions of all variables. Confidence intervals are reported in brackets. *p < 0.10; **p < 0.05; ***p < 0.01.

**Table 5:** Propensity score matching results.

Year	Smart device (ref = No)	Nearest-neighbor			Radius matching			Kernel matching		
		ATT	T	SE	ATT	T	SE	ATT	T	SE
2018	If_smart_device	−0.425	−3.801	0.112	−0.496	−6.102	0.081	−0.495	−6.079	0.081
2018	Any monitoring device	−0.530	−4.886	0.108	−0.538	−6.620	0.081	−0.538	−6.613	0.081
2018	Any assistive device	−0.826	−3.297	0.251	−1.082	−5.922	0.183	−1.078	−5.894	0.183
2018	Any care device	0.517	1.832	0.282	0.372	1.958	0.190	0.404	2.127	0.190
2020	If_smart_device	−0.746	−4.734	0.158	−0.540	−4.399	0.123	−0.539	−4.383	0.123
2020	Any monitoring device	−0.809	−5.056	0.160	−0.616	−5.151	0.120	−0.618	−5.156	0.120
2020	Any assistive device	−0.419	−1.495	0.280	−0.327	−1.575	0.208	−0.336	−1.616	0.208
2020	Any care device	−0.048	−0.207	0.231	0.033	0.203	0.161	0.035	0.219	0.161

The results of sensitivity analyses reported in Tables 4, 5 broadly support the baseline findings that smart device ownership is negatively associated with depressive symptom scores. This pattern is particularly consistent for the breadth of smart device ownership and monitoring device ownership. Although the magnitude and statistical significance of some estimates vary across specifications, the main conclusion remains unchanged. Given the possibility of unobserved confounding, these estimates should be interpreted as robust associations rather than causal effects.

### Mechanism analysis

To investigate the underlying channels through which smart device ownership is associated with depressive symptoms, we conducted a mediation analysis to decompose the total association into direct and indirect components using mediation effect test method (49). Specifically, we test whether social networks, offline activities, and social adaptation serve as mediating channels. This framework assesses whether the association between smart device ownership breadth and depressive symptoms is mediated by these intermediate outcomes. The specific models are as follows (see Equations 6–8):

$$\;\text{Depressio}n_{i}=\alpha_{0}+\alpha_{1}\mathrm{HW}\_\text{tota}l_{i}+\alpha_{2}\text{Contro}l_{i}+\mu_{i}\;\;\;\;\;$$ (6)

$$\text{Mediato}r_{i}=\gamma_{0}+\gamma_{1}\mathrm{HW}\_\text{tota}l_{i}+\gamma_{2}\text{Contro}l_{i}+\delta_{i}$$ (7)

$$\text{Depressio}n_{i}=\rho_{0}+\rho_{1}\mathrm{HW}\_\text{tota}l_{i}+\rho_{2}\text{Mediato}r_{i}+\rho_{3}\text{Contro}l_{i}+\theta_{i}$$ (8)

The term $\text{Mediato}r_{i}$ represents the three mediating variables. $\mu_{i}$ , $\delta_{i}$ , and $\theta_{i}$ are error terms. All models are estimated using pooled OLS with robust standard errors.

As shown in Table 6, the mediation regressions suggest that social network and social adaptation are two key channels linking the breadth of smart device ownership to depressive symptom scores. Table 6 and Appendix Figure A2 presents the estimated mediation paths, and Table 7 reports the bootstrap estimates and confidence intervals for the indirect effects based on 1,000 nonparametric replications. According to the bootstrap results in Table 7, the indirect effect through social networks is −0.024 (95% CI: [−0.031, −0.018]), and the indirect effect through social adaptation is −0.026 (95% CI: [−0.034, −0.017]). These confidence intervals exclude zero, indicating statistically significant mediation through these two channels. In contrast, the indirect effect through offline activity participation is small and statistically insignificant (*β* = −0.006, 95% CI: [−0.018, 0.006]), suggesting that offline activity does not constitute a robust mediation pathway in this sample. The total indirect effect is −0.056 (95% CI: [−0.073, −0.039]), accounting for approximately 23% of the total association. These results indicate that the mediators only partially explain the relationship between the breadth of smart device ownership and depressive symptoms. Social network and social adaptation appear to play more important roles than offline activity participation, suggesting that broader smart device ownership may be associated with lower depressive symptom scores partly through stronger social connections with family members, relatives, and friends, as well as better adaptation to social change.

**Table 6:** The results of mediation effect.

Variables	Social network		Offline activity		Social adaptation		All mediators
	Mediator	Outcome	Mediator	Outcome	Mediator	Outcome	
HW_total	0.443***	−0.217***	0.738***	−0.231***	0.233***	−0.214***	−0.184***
	[0.363, 0.523]	[−0.270, −0.164]	[0.678, 0.799]	[−0.286, −0.176]	[0.160, 0.305]	[−0.266, −0.162]	[−0.237, −0.130]
Social network		−0.051***					−0.055***
		[−0.061, −0.040]					[−0.066, −0.045]
Offline activity				−0.012			−0.008
				[−0.029, 0.005]			[−0.024, 0.009]
Social adaptation						−0.108***	−0.111***
						[−0.120, −0.096]	[−0.123, −0.099]
Indirect (a × b)	−0.022		−0.009		−0.025		−0.056
Direct (c′)		−0.217		−0.231		−0.214	−0.184
Total (c)	−0.240		−0.240		−0.240		−0.240
Control variables	YES	YES	YES	YES	YES	YES	YES
N	12,249	12,249	12,249	12,249	12,249	12,249	12,249
Adjusted R-squared	0.054	0.113	0.218	0.107	0.090	0.132	0.140

Outcome: depressive symptom score (higher = worse). All models include the same control set. See Table 1 for the complete definitions of all variables. Confidence intervals are reported in brackets. The indirect effects in this table are calculated as products of the estimated a and b paths. Bootstrap confidence intervals for the indirect effects are reported in Table 7. *p < 0.10; **p < 0.05; ***p < 0.01.

**Table 7:** Bootstrap mediation confidence intervals.

Effect	Estimate	SE	z	*p*-value	95% CI lower	95% CI upper
Social network	−0.024	0.003	−7.353	0.000	−0.031	−0.018
Offline activity	−0.006	0.006	−0.925	0.355	−0.018	0.006
Social adaptation	−0.026	0.004	−5.915	0.000	−0.034	−0.017
Total indirect	−0.056	0.009	−6.569	0.000	−0.073	−0.039
Direct	−0.184	0.028	−6.650	0.000	−0.238	−0.129
Total	−0.240	0.028	−8.685	0.000	−0.294	−0.185

Bootstrap confidence intervals are based on 1,000 replications.

### Heterogeneity analysis

To examine whether the association between smart device ownership and depressive symptoms varies across population subgroups, we conduct heterogeneity analyses along four dimensions: age, empty-nest status, ADL limitations, and IADL limitations. Empty-nest older adults are defined as those living alone or living only with a spouse. These factors capture important structural differences in life-course stage, living arrangement, and functional capacity, all of which may shape older adults’ demand for, access to, and effective use of smart devices, potentially generating heterogeneous associations with mental health outcomes across groups. The results are shown in Table 8 and Appendix Figures A3, A4.

**Table 8:** Heterogeneity analysis by subgroups.

Variables	Age			Empty-nest		ADL limitation		IADL limitation	
	Age 60–70	Age 71–80	Age >80	Empty-nest = 1	Empty-nest = 0	ADL = 0	ADL > = 1	IADL = 0	IADL > = 1
Panel a: association of any smart device ownership									
If_smart_device	−0.444***	−0.631***	−0.333*	−0.446***	−0.572***	−0.497***	−0.360	−0.530***	−0.344***
	[−0.609, −0.279]	[−0.835, −0.427]	[−0.684, 0.018]	[−0.600, −0.292]	[−0.768, −0.377]	[−0.623, −0.371]	[−0.795, 0.075]	[−0.669, −0.392]	[−0.595, −0.093]
N	6,703	4,072	1,494	7,646	4,623	11,353	916	9,527	2,742
Adjusted R-squared	0.097	0.090	0.069	0.098	0.119	0.090	0.098	0.095	0.103
Control variables	YES	YES	YES	YES	YES	YES	YES	YES	YES
Panel b: association of number of smart devices owned									
HW_total	−0.220***	−0.259***	−0.188**	−0.197***	−0.297***	−0.242***	−0.110	−0.266***	−0.154***
	[−0.295, −0.146]	[−0.347, −0.172]	[−0.340, −0.036]	[−0.268, −0.126]	[−0.378, −0.215]	[−0.299, −0.184]	[−0.251, 0.032]	[−0.330, −0.202]	[−0.251, −0.058]
N	6,703	4,072	1,494	7,646	4,623	11,353	916	9,527	2,742
Adjusted R-squared	0.098	0.089	0.071	0.098	0.122	0.091	0.097	0.096	0.104
Control variables	YES	YES	YES	YES	YES	YES	YES	YES	YES

Outcome: depressive symptom score (higher = worse). All models include the same control set. See Table 1 for the complete definitions of all variables. Confidence intervals are reported in brackets. *p < 0.10; **p < 0.05; ***p < 0.01.

Any smart device ownership and device-ownership breadth are negatively associated with depressive symptom scores across all age groups. Among respondents aged 60–70, 71–80, and above 80, any smart device ownership is associated with 0.444, 0.631, and 0.333 points lower depressive symptom scores (95% CI: [−0.609, −0.279], [−0.835, −0.427], and [−0.684, 0.018]), while each additional smart device owned is associated with 0.220, 0.259, and 0.188 points lower scores (95% CI: [−0.295, −0.146], [−0.347, −0.172], and [−0.340, −0.036]). The negative association is larger among respondents aged 71–80, possibly because health-management and daily-support needs become more salient at this stage of later life. Regarding empty-nest and non-empty-nest groups, the coefficients for any smart device ownership are −0.446 and −0.572 (95% CI: [−0.600, −0.292], [−0.768, −0.377]), while each additional device owned is associated with 0.197 and 0.297 points lower depressive symptom scores (95% CI: [−0.268, −0.126], [−0.378, −0.215]). This pattern may reflect the role of co-resident family members in facilitating device access and helping older adults integrate smart devices into daily routines.

Functional limitations show more pronounced heterogeneity in the subgroup-specific estimates. Among respondents without ADL limitations, any smart device ownership is associated with a 0.497 points lower depressive symptom score (95% CI: [−0.623, −0.371]), while each additional smart device owned is associated with a further 0.242 points lower score (95% CI: [−0.299, −0.184]). By contrast, among respondents with ADL limitations, the corresponding estimates are smaller and statistically insignificant. The results of IADL subgroup show a similar pattern. Among respondents without IADL limitations, any smart device ownership is associated with a 0.530 points lower depressive symptom score (95% CI: [−0.669, −0.392]), and each additional device owned is associated with a 0.266 points lower score (95% CI: [−0.330, −0.202]). Among those with IADL limitations, the corresponding associations remain negative but are weaker, at −0.344 and −0.154, respectively (95% CI: [−0.595, −0.093], [−0.251, −0.058]).

## Conclusion and discussion

### Main conclusion

This study expands the limited empirical evidence on the association between smart device ownership and depressive symptoms among older adults in China, using data from the 2018 and 2020 waves of CLASS. We found that smart device ownership is generally associated with fewer depressive symptoms among older adults, which is consistent with existing research related to digitalization and older adults’ mental health (28, 29, 41). However, the association observed in this study varies across device functions and specific device types, suggesting that its mental health relevance depends on the functions they serve and the needs associated with ownership. Heterogeneity analyses further show that this association is weaker among older adults with functional limitations.

Our findings suggest that digitalization and technological advancement may have positive implications for the aging population, but these implications are likely to depend on the match between device functions and the owner’s health and functional needs. Monitoring and assistive devices are more consistently associated with fewer depressive symptoms, possibly because they are mainly related to routine health management, daily functioning, and independent living, whereas the positive association for care devices may reflect poorer physical conditions and greater care needs among their owners rather than an adverse effect of the devices themselves. The contrasting patterns within care device ownership further suggest that the diffusion of digital technologies does not generate equal mental health benefits for all older adults. Without better functional adaptation, usability, and support for vulnerable owners, smart technologies may deliver limited benefits to those with special care needs and may unintentionally reinforce pre-existing inequalities in later life. Specifically, smart cameras may be linked to perceived safety and reflect caregiver attention, which could explain their weak negative association with depressive symptoms (50). In contrast, although smart speakers may potentially provide information access and communication functions, they are better understood as complementary tools with operation barriers rather than substitutes for family companionship or direct social interaction (51). Thus, the positive association observed for smart care device category should be interpreted as reflecting potential care needs of their owners.

The mediation results indicate that social network and social adaptation, as relational and adaptive channels, outweigh general offline activities in explaining the association. These results suggest that broader smart device ownership is more strongly associated with lower depressive symptom scores when it is accompanied by stronger social ties, greater perceived connectedness, and better adaptation to a rapidly changing social environment. This pattern is consistent with the view that digital technologies may improve older adults’ well-being by facilitating social integration and reducing social exclusion in later life (18, 26, 34). By contrast, the insignificant channel related to offline activities suggests that the psychological relevance of smart devices does not rely simply on increasing activity frequency, but rather on whether device ownership is linked to meaningful social connection and adaptive capacity.

### Limitations and future research

This study has several limitations that should be acknowledged.

First, the measurement of smart device is constrained by the questionnaire. The CLASS survey records ownership of specific smart devices, but does not provide detailed information on actual use, including frequency, duration, purpose, or proficiency. Therefore, this study uses device ownership as core explanatory variable to exam the association between smart devices and depressive symptoms. This variable is imperfect but remains meaningful because the devices examined are function-specific products that are usually acquired to meet particular needs. For example, electronic blood pressure monitors are typically purchased for regular health tracking, smart wheelchairs for mobility support, and smart cameras for home monitoring and emergency detection. Future research should collect more detailed information on actual usage behavior, purposes, and use contexts.

Second, although we include extensive controls and conduct sensitivity analyses, unobserved confounding may still remain, partly because the survey lacks detailed measures of digital literacy, mental health service use, physical activity, and actual device-use contexts. Therefore, the findings should be interpreted cautiously as associations rather than causal effects.

Third, this study uses the latest publicly available CLASS data up to 2020. Although the data do not capture the recent diffusion of AI-enabled care technologies, they provide baseline evidence for future research to examine whether newer digital technologies reshape the associations observed in this study.

### Policy implications

This study yields several findings that carry important policy implications.

First, digital inclusion policies should focus not only on the access to smart devices, but also on improving device-related services by simplifying interfaces, developing age-friendly functions such as voice recognition, and encouraging devices with diverse prices range and stronger health-related functions, thereby helping smart devices better support health management, functional independence, and social connectedness among older adults.

Second, the producers should avoid undifferentiated smart device design and instead adopt a function-oriented approach to match technologies with older adults’ health conditions, functional capacity, and care contexts. Monitoring devices should prioritize data accuracy, simple operation, age-friendly interfaces, clear feedback, and links with primary healthcare services to support routine health management. Assistive devices should emphasize functional reliability, and integration into daily living environments to help older adults maintain independence and reduce functional barriers. Smart care devices should be integrated with family, community, and long-term care support, with caregivers and service providers helping older adults interpret information, respond to alerts, and obtain practical and emotional assistance.

Third, policies should strengthen the social and adaptive channels linking smart device ownership to later-life mental health by integrating family support, community-based services, and professional long-term care institutions. Since offline activity is not a robust mediation pathway, policies should focus less on simply increasing activity frequency and more on improving its social quality and psychological relevance.

Fourth, older adults with functional limitations may face greater difficulty in converting device ownership into actual use and mental health benefits. For this group, policies should place greater emphasis on assisted use, routine integration, and care coordination through family caregivers, community workers, and primary healthcare providers.

## Data Availability

Publicly available datasets were analyzed in this study. This data can be found at: http://jkzgyjy.ruc.edu.cn/sjzy/CLASSsjsq/index.htm.
