# Supplementary material for: Smart device ownership and depressive symptoms among older adults: evidence from China
Source: Front Public Health. 2026 Aug 6;14:1881246. doi: 10.3389/fpubh.2026.1881246 (PMC13489914; doi:10.3389/fpubh.2026.1881246)
Supplement: Supplementary file 1 [file Supplementary_File_1.docx]

**Appendix**

**Figure A1 Association between smart device ownership and depressive symptoms**

*Note*: Estimates are from the corresponding models in Table 3

**Figure A2 Mediation analysis results.**

*Note:* Point estimates and bootstrap 95% confidence intervals are based on 1,000 replications.

**Figure A3 Heterogeneity in smart device association by subgroup (Panel a: Any smart device ownership)**

*Note:* Coefficient and 95% confidence intervals are from subgroup-specific OLS regression in Table 8 Panel a.

**Figure A4 Heterogeneity in smart device association by subgroup (Panel b: Breadth of smart device ownership)**

*Note:* Coefficient and 95% confidence intervals are from subgroup-specific OLS regression in Table 8 Panel b.
